# System-level policies on appropriate opioid use, a multi-stakeholder consensus

**DOI:** 10.1186/s12913-022-07696-x

**Published:** 2022-03-12

**Authors:** Patrice Forget, Champika Patullo, Duncan Hill, Atul Ambekar, Alex Baldacchino, Juan Cata, Sean Chetty, Felicia J. Cox, Hans D. de Boer, Kieran Dinwoodie, Geert Dom, Christopher Eccleston, Brona Fullen, Liisa Jutila, Roger D. Knaggs, Patricia Lavand’homme, Nicholas Levy, Dileep N. Lobo, Esther Pogatzki-Zahn, Norbert Scherbaum, Blair H. Smith, Joop van Griensven, Steve Gilbert

**Affiliations:** 1 Institute of Applied Health Sciences, Epidemiology Group, School of Medicine, Medical Sciences and Nutrition, University of Aberdeen, Foresterhill, AB25 2ZD Aberdeen, UK; 2 Department of Anaesthesia, NHS Grampian, Aberdeen, AB25 2ZD UK; 3 Pharmacy Department, Queensland Opioid Stewardship Program, Clinical Excellence Queensland, Royal Brisbane and Women’s Hospital, Herston, Queensland 4069 Australia; 4 NHS Lanarkshire, Motherwell, ML1 2TP UK; 5 National Drug Dependence Treatment Centre and Department of Psychiatry, All India Institute of Medical Sciences, New Delhi, 110029 India; 6 International Society of Addiction Medicine (ISAM), Valletta, Malta; 7 Psychiatry and Addictions, University of St Andrews, St Andrews, Scotland; 8 NHS Fife Addiction Services, Fife, KY16 UK; 9 MD Anderson Cancer Center, University of Texas, Houston, TX 77030 USA; 10 Department of Anaesthesiology and Critical Care, Faculty of Medicine and Health Sciences, Cape Town, 8000 South Africa; 11 Pain Management Service, Royal Brompton & Harefield NHS Foundation Trust, London, UK; 12 Department of Anesthesiology, Pain Medicine and Procedural Sedation and Analgesia, Martini General Hospital Groningen, van Swietenplein 1, 9728 NT Groningen, The Netherlands; 13 Chronic Pain, Modernising Patient Pathway Programme, Scottish Government, Edinburgh, UK; 14 Calderside Medical Practice, Blantyre, South Lanarkshire G72 0BS Scotland, UK; 15 University of Antwerp (UAntwerp, CAPRI), Antwerp, Belgium; 16 Psychiatric Center Multiversum, 2530 Boechout, Belgium; 17 Centre for Pain Research, The University of Bath, Bath, BA2 7AY UK; 18 UCD School of Public Health, Physiotherapy and Sports Science, University College Dublin, Dublin, Ireland; 19 Pain Alliance Europe, Rue de Londres 18, 1050 Brussels, Belgium; 20 School of Pharmacy, University of Nottingham, University Park, Nottingham, NG7 2RD UK; 21 Anesthesiology Department, Cliniques universitaires Saint-Luc, UCLouvain, 1200 Brussels, Belgium; 22 West Suffolk Hospital, Bury St. Edmunds, IP33 2QZ UK; 23 Gastrointestinal Surgery, Nottingham Digestive Diseases Centre and National Institute for Health Research Nottingham Biomedical Research Centre, Nottingham University Hospitals NHS Trust and University of Nottingham, Queen’s Medical Centre, Nottingham, NG7 2UH UK; 24 University Hospital of Muenster, Muenster, Germany; 25 LVR-Hospital Essen, Department of Psychiatry and Psychotherapy, Medical Faculty, University of Duisburg-Essen, Essen, Germany; 26 Ninewells Hospital and Medical School, NHS Tayside, University of Dundee, Dundee, DD2 4BF Scotland, UK; 27 Belford Hospital, Fort William, PH33 6BS Scotland, UK

## Abstract

**Background:**

This consensus statement was developed because there are concerns about the appropriate use of opioids for acute pain management, with opposing views in the literature. Consensus statement on policies for system-level interventions may help inform organisations such as management structures, government agencies and funding bodies.

**Methods:**

We conducted a multi-stakeholder survey using a modified Delphi methodology focusing on policies, at the system level, rather than at the prescriber or patient level. We aimed to provide consensus statements for current developments and priorities for future developments.

**Results:**

Twenty-five experts from a variety of fields with experience in acute pain management were invited to join a review panel, of whom 23 completed a modified Delphi survey of policies designed to improve the safety and quality of opioids prescribing for acute pain in the secondary care setting. Strong agreement, defined as consistent among> 75% of panellists, was observed for ten statements.

**Conclusions:**

Using a modified Delphi study, we found agreement among a multidisciplinary panel, including patient representation, on prioritisation of policies for system-level interventions, to improve governance, pain management, patient/consumers care, safety and engagement.

## Introduction

Scientific understanding of opioid pharmacology and of the neurophysiology of pain has been a success story of the twentieth century. In the face of gross global inequalities in access to opioid medications, over the last 30 years, there has been increasingly liberal prescribing of opioids in some parts of the world, both for acute and for persistent or chronic pain [1].

In some countries more than in others, over-supply and overuse resulted in unwanted adverse effects, dependence, addiction, opioid use disorders and overdose-related deaths, which have reached epidemic proportions [2–4]. At the same time, there are problems of access to pain relief in general, including opioids and non-opioids, in many lower income countries.

These issues are relevant at an individual, as well as societal levels, involving public, healthcare professionals and industry, because they find their origins at different levels [5–10]. Unfortunately, although there is increasing recognition of problems occurring with opioid prescribing, as well as the unclear analgesic benefit, even in the acute pain setting, there continues to be high rates of harm from opioid prescribing and no universal policy orientation [11, 12].

Therefore, consensus is needed, not only on the prescribing of opioids, but also on policies for system-level interventions, including governance, pain management, patient care and patient engagement [13, 14].

Such a consensus requires collaboration between different disciplines: pain specialists, anaesthesiologists, surgeons, general practitioners, nurses, psychologists, pharmacists, addiction specialists and educators. In addition, healthcare organisations and governmental bodies will need to support the development of a culture of more rational and safe prescribing. This may include prescribing guidelines such as limitations on medication types, dosage, and duration, as well as patient education, provider training, clear referral mechanisms, and other public health initiatives such as real-time monitoring of prescribing. The public and patients should be involved in the context of informed consent and shared decision-making.

Whilst a review of clinical practices has recently been undertaken [15], policies at institutional and societal (governmental and healthcare system) levels have not. The current work aims to identify where there is consensus regarding these policies.

## Methods

### Methodology model

A modified Delphi method was used. This technique was selected because it has been used widely to generate robust consensus in healthcare research and does not require face-to-face contact. It has proven very useful in related projects [16].

### Panellist recruitment

The steering committee recruited panellists by inviting members of major scientific societies (European Pain Federation EFIC, International Association for the Study of Pain IASP, International Society of Addiction Medicine ISAM, Pain Alliance Europe PAE, European Federation of Addiction Societies EUFAS, European Psychiatric Association EPA) and their networks. This ensured all relevant profiles were represented i.e. involved in pain and/or in addiction problematics. Along with healthcare representation (different medical disciplines namely anaesthesia/pain medicine, addiction medicine/psychiatry, surgery, nursing, physiotherapy and pharmacy) consumers (patient representatives) were also included in the panel (Table 1).

**Table 1:** Participant profiles

Profile	Number (total = 23)
Consumer Representatives	2
General Practitioners	2
Nurse (specialised in Pain Management)	1
Pharmacists (specialised in pain, substance misuse)	2
Physicians specialised in Anaesthesia and/or Pain Management (acute and/or chronic)	9
Physicians specialised in Addiction Medicine/Addiction Psychiatry	4
Physician specialised in Surgery	1
Physiotherapist specialised in Pain management	1
Psychologist specialised in Pain management	1
**Country**	
Australia	1
Belgium	3
Finland	1
Germany	2
India	1
Ireland	1
South Africa	1
The Netherlands	1
United Kingdom	11
USA	1

Given the range of perspectives on pain and its management in different social, professional and geographical settings, we also invited, and included participants from Europe, Asia, Africa, Oceania and America.

### Policies on appropriate opioids use

Policies were developed by the steering group, based on the current literature on quality improvement using Pain Management Stewardship groups [17, 18]. A first version of the statement was drafted by the steering committee, and then presented at International Society of Addiction Medicine—Canadian.

Society of Addiction Medicine Joint Conference 2020 (12th to 14th November 2020), Victoria, BC, Canada. All statements were presented and comments were taken into account to finalize the proposed statements in the first round.

Governance policies, pain management policies and policies regarding patient care and consumer engagement are detailed, in their final version, in the Table 2.

**Table 2:** Results of the Delphi survey. Statements are in their final version

Statements	Agreement on the final version	Changes	Agreement on prioritisation	Changes
**Governance policies**				
**The presence of a Pain Management, Analgesia or Opioid Stewardship Steering Committee, with multidisciplinary representation from Key Stakeholders is a priority in the context of acute pain, especially in the hospital.** Key goals of this committee will be to inform, coordinate and action tasks and projects which support comprehensive Stewardship, supported by local data.	**91%**	**+ 11%**	**96%**	**+ 1%**
**The health system should have policies in place which outline the safe and accountable use (closed-loop) of drugs which could lead to dependence and/or unnecessary morbidities or mortalities, such as opioids, combined or not with gabapentinoids, cannabinoids.** This includes policies and practices around storage (including strategies to avoid selection error), order, transfer, administration (including comprehensive independent second checks) and disposal of controlled drugs within the hospital, management and return of patient’s controlled drugs, and strategies to identify and address diversion. This should be context and country sensitive.	**100%**	**+ 5%**	**96%**	**+ 11%**
**Pain Management policies**				
**Research and policies should be developed to have a better understanding and vision of who are the prescriber and what are the determinants of the prescribing practices, not only about opioids but also about non-opioid and non-pharmacological pain management strategies.** This could include research about local strategies, development of specific pain management guidelines, educational programmes, agreement to the use of Therapeutic Guidelines, additional structure around referral criteria (‘Traffic Light’ System as often used for Antimicrobial Prescribing), analgesia de-escalation guidelines, discharge prescribing guidelines.	**100%**	**+ 5%**	**91%**	**+ 6%**
**Policies should be developed providing guidelines on maximum doses and duration of treatment for high-risk medications such as opioids and high-risk combinations.** While most opioid medications will not necessarily have a ‘maximum’ licenced dose, it should be agreed the dose and duration at which senior or specialist review or approval is required.	**86%**	**+ 1%**	**76%**	**−6%**
**Processes and guidelines should be prioritised regarding the quality of the follow-up and the referral on high-risk patients.** This may include referral pathways in pre-admission clinics for patients on > 50 mg oral morphine equivalent daily dose (OMEDD). Doses of these high-risk medications should always be confirmed with prescriber or dispensing pharmacy.	**87%**	**+ 2%**	**78%**	**+ 3%**
**Every hospital should have a programme for identifying the places where opioids are used systematically and identifying whether any regular re-assessment based on relevant** **outcomes occur.** This would aim to identify, and sensitize the prescribers, when opioids are systematically used without any relevant evidence, before surgery, during the perioperative period or prescribed systematically at the hospital discharge.	**96%**	**+ 1%**	**91%**	**+ 6%**
**There is a specific hazard with adding new constraints on opioid prescribing that should be specifically addressed to prevent the risk of leading to the pendulum swinging too far in the other direction, with the risk of patients suffering unnecessarily pain possibly responsive to opioids.** The place of opioid should be determined, i.e. where and when they are/will be identified as highly effective and without better alternative so as not to create counterproductive measures.	**100%**	**0%**	**96%**	**+ 4%**
**Patient Care and Consumer Engagement**				
**Processes should be developed to facilitate and monitor the return any unused or expired analgesic medications or promote the nearest pharmacy which will accept returns.** This may include promotion in patient counselling materials, in pharmacy signage, or in local publications such as newspapers, websites or social media accounts.	**91%**	**+ 6%**	**52%**	**−3%**
**Every system should develop mechanisms to regularly audit to monitor, disseminate, and benchmark indicators of the quantity and appropriateness of opioid use and quality of pain management.** This may include a real-time monitoring, specific to acute and/or postoperative pain, including the analysis of combination with other hypnotics like gabapentinoids, cannabinoids or benzodiazepines.	**100%**	**+ 10%**	**96%**	**+ 1%**
**Public opinion and societal attitudes should be explored on the implications of ‘Pain relief as a human right’.**	**86%**	**+ 21%**	**59%**	**+ 4%**
**Secondary care practitioners should identify and provide opportunities to interact with General Practitioners and other community providers in promoting pain management and** **opioid/gabapentinoid/benzodiazepines stewardship after prescription for acute pain.** Secondary care may provide guidance on duration of analgesia prescriptions and there should also be opportunities for primary care to feedback on the suitability of guidance. This may include invitations to relevant Grand Rounds presentations, or organising forums with local community providers to discuss pain management and opioid stewardship, Q & A evenings, seeking feedback regarding discharge handover, engagement through GP Liaison Officers.	**100%**	**+ 10%**	**96%**	**+ 6%**
**Support and access to primary or secondary care deaddiction services should be available to patients who develop substance use disorder as a result of prescribed analgesia.** This may include the development of multidisciplinary ‘Pain and Dependency’ services integrating psychosocial and medical care.	**96%**	**+ 1%**	**100%**	**0%**
**Information from drug intelligence and law enforcement agencies as well as from overdoses and deaths related to prescribed drugs should be fed back to prescribers.**	**87%**	**NA**	**65%**	**0%**

### Data collection structure

After compiling a group representing all the desired expertise, 25 panellists in total were invited using a secured web-based platform. The responses were collected and managed using REDCap (Research Electronic Data Capture) electronic data capture tools [19]. REDCap is a secure, web-based application designed to support data capture for research studies, providing 1) an intuitive interface for validated data entry; 2) audit trails for tracking data manipulation and export procedures; 3) automated export procedures for seamless data downloads to common statistical packages; and 4) procedures for importing data from external sources. This ensured the anonymity of data collection, the structuring and controlling the data capture and providing feedback to the panellists (namely the automatization of the report of the response from one round to the subsequent one).

### Data collection process

In round 1 the participants were asked to rate the 12 statements drafted by the steering committee, based on a scoping review of the literature using a five-levels Likert scale. They also had the opportunity to propose new statements.

In subsequent rounds, participants had the option to change their rating after seeing their selection with regard to the (aggregated and anonymised) selections of the other voters agreeing/disagreeing. In rounds 3 and 4 the items for which an agreement had been reached were presented to determine whether or not this should be prioritised (yes/no).

### Data analysis

An aggregation of responses was semi-automated using REDCap and available for the participants in total. For the final report presented here, an aggregate of strongly agreed and agreed was used to determine if 75% threshold for agreement was met. A high stability was defined as < 10% changes in absolute proportions between the last round and the previous one (looking at the construct or at the prioritisation of it).

### Ethical review

Aligned with United Kingdom (UK) Policy Framework for Health and Social Care Research, this study has been granted an exemption from requiring ethics approval (Name of the ethics committee: NHS Research Ethics Committee, REC), according to the Central Office for Research Ethics Committees (COREC) and the National Research Ethics Service (NRES) guidance. Written informed consent was obtained from all participants.

## Results

### Round 1

Twenty-three of the 25 invited panellists participated in the Delphi survey. Two who initially accepted the invitation to be part of the study did not respond to subsequent emails. Participants, with varied professional background and roles, came from primary, secondary care and academia, from secondary and tertiary referral hospitals (Table 1).

Other than the 2 consumer representatives, all participants were regularly involved in providing direct patient care.

Twelve items were proposed to the participants. All 23 participants completed the 12 items survey and 12 added comments.

### Round 2

All 23 participants from round 1 participated in round 2.

Based on the panellists feedback in round 1 six of the 12 items were modified according to the participant’s suggestions in round 1 and a 13th statement (item) was added: ‘Information from drug intelligence and law enforcement agencies as well as from overdoses and deaths related to prescribed drugs should be fed back to prescribers’ . The modifications are detailed in Table 2. All were discussed by the steering committee.

All 13 statements reached the predefined agreement threshold (75%) and only one statement did not meet the definition of high stability (< 10%) changes, but as this change was + 11% of additional agreement, we decided to proceed to Round 3 (Table 2). The final versions are presented in this document.

### Rounds 3 and 4

All 23 panellists completed the prioritisation questionnaires in due time. The 75% threshold for agreement was reached to prioritise the statements for all but for three: *Processes should be developed to facilitate and monitor the return of any unused or expired analgesic medications* (52%); *Public opinion and societal attitudes should be explored on the implications of ‘Pain relief as a human right’* (59%); *Feedback from drug intelligence and law enforcement agencies* (65%).

Stability was high for all but for one: *The health system should have policies in place which outline the safe and accountable use (closed-loop) of drugs* (+ 11%).

Where an agreement was reached, it related to statements (Table 2) grouped afterwards in themes. This related to information, coordination and implementation tasks by regulatory authorities; promotion, monitoring and support of safe and appropriate prescribing; 2. Better understanding of prescribing practices; 3. Communication, follow-up, referral and patient-centredness. The agreement varied between 76 and 100% with a percentage change between the last two rounds ranging from − 6% to + 11%. Concrete propositions were made (Table 2).

## Discussion

This Delphi survey reached a robust agreement on thirteen system-level policy statements on the appropriate use of opioids. Ten of them were considered priorities by the panellists. These statements related to improving governance, pain management, patient/consumers care, safety and engagement.

### What are the priorities in terms of governance policies?

This work highlights the needs for developing Pain and Opioid Stewardship Steering Committees. These committees could facilitate the implementation of policies and best practices for the safe and responsible use of opioids or other drugs that could lead to dependence. According to the panellists, this should be sensitive to the country and could be implemented at both national levels and hospital/healthcare system levels. Such a committee may supervise Opioid Stewardship programmes.

The Institute for Safe Medication Practice Canada describes Opioid Stewardship as “coordinated interventions designed to improve, monitor, and evaluate the use of opioids in order to support and protect human health”. By an interprofessional, multidisciplinary approach, the goal is to optimise opioid prescribing while minimizing unintended consequences. This could be elegantly integrated into metrics as quality indicators, rather than just looking at the number of prescriptions. But this steering committee could also look at other solutions that have been proposed for chronic or persistent pain, like:

- Education: Increasing the importance of pain management education [20].
- Individualised prescribing: Rather than indiscriminately reducing supply, perhaps the focus should be on tailoring pain medication prescriptions to the individual, such as tailoring the number of pills to need.
- Monitoring prescriptions: continuing prescriptions and repeat prescribing after surgery have been shown to be a significant factor in persistent opioid use. Reviewing the effectiveness of analgesia and the ongoing need should be recognised as a normal part of good medical practice.
- Reducing risk; identification of risk factors for predicting the development opioid use disorders are limited in their accuracy [21] but must be part of the prescription process, with an appropriate informed consent [22].
- Opioid reduction programmes: helping patients to gradually reduce opioids has been shown to be achievable and to not lead to worsening of pain. Indeed, patient quality of life has been found to be improved [23].

However, in addition to the complexity of deprescribing, the committee recognizes that there is a risk that overreaction and regulation of opioid prescribing could cause the pendulum to tip too far in the other direction, with the possibility that patients may suffer needlessly [24, 25].

### How to improve pain management (policies)?

As opioids play a critical role in pain management, especially after surgery and trauma, it is necessary to achieve a balance between providing adequate analgesia while minimising the risk of medication related harm. Evidence shows that the risk of long-term opioid use increases with each additional day of the initial prescription supply, [26] particularly if more than 5 days of therapy is prescribed [27]. This supports the development of strategies aiming to manage persistent pain in the weeks after surgery, as well as persistent opioid use, stewarding the patient by a careful tailored approach.

Further research is also needed. Even after decades of investigations, the transition from acute to chronic pain remains poorly understood [28–32]. One important factor that has become evident is the potential for opioids to cause acute opioid-induced hyperalgesia (playing a potential role in the sensitization to pain of the central nervous system), and persistent postoperative opioid use, associated with a significant risk for the patient, with unclear and inconsistent evidence for benefit in relieving pain [33].

### Patient care and consumer engagement

Clearly, anaesthetic, analgesic and surgical plans should be constantly questioned, and integrated into a holistic vision; patient-centred optimisation of pre- and perioperative management, including screening for vulnerability factors, not only physical, but also psychosocial factors. Reinforcing and structuring communication plans during the perioperative phase, between secondary care prescribers, acute pain services and primary care is clearly highlighted by this consensus. The Faculty of Pain Medicine UK Best Practice Guidelines on Opioids and Surgery [34] recommend assessment and management by chronic as well as acute pain management services, for patients undergoing surgery to facilitate specific measures, such as reducing opioid use in the preoperative period, assessing the appropriateness of gabapentinoid prescription and addressing psychosocial factors which are associated with increased risk of poorly controlled postoperative pain, persistent postoperative pain and opioid use. Good practices should be facilitated such as the return of unused medicines and, in general, provide specific and appropriate information and trigger patient and consumer engagement. Improving communication (verbal and written) will enhance patients understanding of pain. Pre-operative information and education that incorporate a health literacy sensitive approach (use of plain language, talk back strategy – asking patient to repeat back what has been said to them to ensure understanding) would ensure a patient centred approach to pain management. Informed consent to treatment could improve patient experience, reduce risks and harms – to the patient and within society [35]. Finally, links with addiction services to inform assessment and risk management would contribute to minimising the likelihood of patients developing problematic drug use. There should be a referral process for high-risk patients and access to pain and dependency services.

### What can we learn from current practices, guidelines and policies?

In the last 10 years, prescription rates of strong opioids have more than doubled in most developed countries. North America, Europe and Australasia have seen the greatest increases. The widespread use of opioids and its association with premature mortality demonstrate that this is a major public health issue. Guidelines, mostly at the intention of prescribers have been developed, for instance in the UK, as there is a need to develop consistent evidence on effective and safe prescription strategies [3, 28, 36–38]. But evidence is lacking regarding the effect, at the system level, of policies on prescriptions and the different strategies adopted internationally.

Prescription patterns are best described in North America and Australia, including specific, potentially modifiable, factors [39]. But little is known about the optimal pathways (acute, episodic and chronic; after surgery; risky co-prescriptions; relationships with socio-economic factors and mortality). This could be addressed by the development of real-time prescription monitoring, permitting us to study and to identify sources of variability and opportunities for improvement. This would allow us to identify where problems may exist while protecting patients from a blind approach based solely on reducing prescription rates. Concrete propositions are made in Table 2.

The findings in this research reflect the views of this multi-stakeholder panel. As a result, their specific perspectives and experiences are a limitation of a Delphi-based consensus. However, the panel was recruited to represent a breadth of perspectives to improve the robustness of the work. Additionally, the strength of the consensus suggests that the final statements are at least partially, if not fully, translatable to most contexts. Finally, although the Delphi method is good for reaching consensus, it may also moderate the findings.

## Conclusion

The opioid epidemic has been a wake-up call for medicine to take stock of how pain is managed. Unintended consequences of persistent opioid use and related harm have been observed. When putting system-level policies in place to improve pain management, we must also design safeguards that will reduce the risk of overprescribing, while encouraging a more biopsychosocial approach.

In this Delphi study, we have identified themes and priorities relating to concrete actions for regulatory authorities. We have found agreement to prioritize policies for system level interventions, to improve governance, pain management, patient / consumer care, safety and engagement. In terms of governance, this includes projects to promote, monitor and support the safe and appropriate prescribing of drugs which often lead to dependence, such as opioids. For pain management policies, a better understanding of prescribing practices and future programs could include guidelines and educational activities to implement them, but also communication, monitoring and referral between 1st and 2nd line caregivers. Finally, patients must be well informed and provided structured/integrated support, including pain management and/or opioid stewardship, in addition to addiction management, when required.

The problem is global, even if the solutions may be different, depending on context and country specific factors. However, common denominators may exist, such as monitoring of practices and improved observational studies.

## Data Availability

All the data generated and analysed during the current study are available in the manuscript.
